# Large cell neuroendocrine carcinoma in pancreatoblastoma with TP53 and SMAD4 mutations: a clinicopathologic study of a rare entity

**DOI:** 10.1093/jscr/rjae654

**Published:** 2024-11-01

**Authors:** Ifeomachukwu E Nwosu, Jenny Zhang, Alexis S Elliott, Michelina De La Maza, Belinda L Sun

**Affiliations:** Department of Pathology, Banner-University Medical Center, College of Medicine, University of Arizona, 1625 N Campbell Ave, Tucson, AZ 85721, United States; Department of Pathology, Banner-University Medical Center, College of Medicine, University of Arizona, 1625 N Campbell Ave, Tucson, AZ 85721, United States; Department of Pathology, Banner-University Medical Center, College of Medicine, University of Arizona, 1625 N Campbell Ave, Tucson, AZ 85721, United States; Department of Pediatrics, Pediatrics Hematology and BMT, Banner-University Medical Center, College of Medicine, University of Arizona, 1625 N Campbell Ave, Tucson, AZ 85721, United States; Department of Pathology, Banner-University Medical Center, College of Medicine, University of Arizona, 1625 N Campbell Ave, Tucson, AZ 85721, United States

**Keywords:** pancreatoblastoma, neuroendocrine carcinoma, pancreatic tumor

## Abstract

Pancreatoblastoma, a rare pancreatic tumor, exhibits diverse differentiation pathways, including acinar, ductal, and neuroendocrine lineages, often with distinct squamoid nests [3]. We present a notable case of pancreatoblastoma coexisting with large cell neuroendocrine carcinoma in a 10-year-old boy, presenting with abdominal discomfort, weight loss, and lesions in the pancreas, spleen, and liver visible on imaging. A liver biopsy revealed a poorly differentiated carcinoma with neuroendocrine features, while a splenic biopsy showed acinar cell differentiation, raising possible diagnoses of pancreatoblastoma or acinar cell carcinoma. Subsequent surgical resection after chemotherapy revealed diverse components within the pancreatoblastoma, including well-differentiated acinar and neuroendocrine cells, squamoid nests, and a high-grade neuroendocrine carcinoma. Genetic analysis detected pathogenic variants in TP53 and SMAD4, rarely found in pancreatoblastomas. This juxtaposition of large cell neuroendocrine carcinoma and pancreatoblastoma suggests a potential evolution from well-differentiated neuroendocrine tumors to poorly-differentiated carcinomas within pancreatoblastomas.

## Introduction

Pancreatoblastoma is a rare malignant neoplasm with an annual incidence of 0.004 per 100 000, with ~200 cases reported in the literature [1, 2]. Neuroendocrine tumors in children are also rare, with an incidence of 2.8 cases per million and a prevalence of 7724 cases reported from 1975 to 2006 [1, 2]. Pancreatoblastoma typically exhibits at least two lines of differentiation: ductal, acinar, or neuroendocrine, and obligatorily includes squamoid nests [3]. The neuroendocrine component of pancreatoblastoma is usually well-differentiated without high-grade features. High-grade neuroendocrine carcinomas within pancreatoblastoma have not been previously described in children. This report presents a case of a 10-year-old boy with a large pancreatic pancreatoblastoma diagnosed with high-grade progression to large cell neuroendocrine carcinoma (LCNEC). Genetic sequencing revealed unique mutations in TP53 and SMAD4, which were not previously reported in pancreatoblastoma but commonly seen in poorly differentiated neuroendocrine carcinomas and pancreatic ductal carcinomas [4, 5].

## Case report

### Clinical history

A 10-year-old boy presented with a one-month history of worsening right upper quadrant and epigastric pain, decreased appetite, weight loss, occasional vomiting, and diarrhea. Physical examination revealed tachycardia and hepatosplenomegaly (liver and spleen, 4 cm and 3 cm below the costal margin, respectively). Laboratory tests showed elevated alpha-fetoprotein (AFP) levels of 357 ng/ml (normal <8.0 ng/ml), with normal beta- Human chorionic gonadotropin and carcinoembryonic antigen (CEA). Magnetic resonance imaging (MRI) of the abdomen/pelvis showed a solid, enlarged, heterogenous splenic mass measuring 10.1 × 7.0 × 9.3 cm, left hepatic lobe lesions with central scars measuring 4.3 × 5.3 cm and 5.3 × 3.7 cm. Computed tomography (CT) of the abdomen/pelvis revealed numerous hepatic nodules and complex cystic/solid pancreatic lesions. A biopsy of the hepatic lesion showed high-grade primitive neoplastic cells forming small acinar structures. IHC demonstrated strong positivity for CK Oscar, nuclear beta-catenin, EMA, and synaptophysin. Ki-67, a proliferation index, was 80%–100%. The diagnosis was poorly differentiated carcinoma with a possibility of neuroendocrine differentiation. The patient underwent six cycles of cisplatin and etoposide chemotherapy according to National Comprehensive Cancer Network (NCCN) guidelines.

Follow-up CT showed minimal improvement in hepatic lesions and splenic masses. A repeat biopsy of the splenic mass exhibited a morphological overlap with the hepatic biopsy but included low-grade components, with a Ki67 index of 1% and acinar differentiation showing focal positivity for trypsin and chymotrypsin. Molecular testing of the splenic mass detected pathogenic mutations in TP53 (exon 5 c.376-1G > A, 11%) and SMAD4 (exon 9, c1052A > G, 43%), along with a PALB2 mutation variant (exon 12, c3249G > C, 29%) of uncertain significance. Tumor Mutation Burden was low. Cancer Type ID molecular testing showed a 90% match with islet cell (neuroendocrine) carcinoma. Based on the morphology, immunoprofile, and genetic tests, two potential diagnoses were considered: pancreatoblastoma versus pancreatic acinar cell carcinoma. The patient underwent chemotherapy with FOLFIRI, and after 10 cycles, rising AFP levels prompted exploratory laparotomy, splenectomy, distal pancreatectomy, and wedge resection of the liver’s third segment. Post-operatively, the patient completed five cycles of folinic acid, fluorouracil and irinotecan hydrochloride (FOLFIRI), five cycles of vincristine, doxorubicin and cyclophosphamide (VDC), three cycles of ifosfamide, carboplatin and etoposide (ICE), and stereotactic body radiotherapy. Due to rising AFP levels raising concern for chemotherapy resistance, he was switched to gemcitabine. The latest AFP level is 379 ng/ml, measured 11 months post-surgery.

### Gross pathology

**Figure 1:**
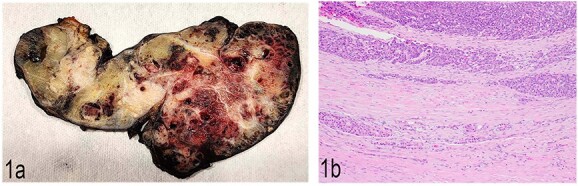
(a) Gross anatomy of the distal pancreas and spleen involved by the mass. (b) Well-differentiated acinar cells, well-differentiated neuroendocrine tumor and LCNEC, H & E, 2×.

Gross examination of the distal pancreatectomy and splenectomy specimen revealed a 16.8 cm large lobulated, circumscribed tumor extensively involving the entire distal pancreas and extending through the splenic hilum into the spleen. The cut surface was heterogenous with pale to tan-yellow areas, focal hemorrhage, and necrosis (Fig. 1a). Multiple nodules were identified in the spleen hilum and multiple vessels were grossly involved by the tumor. The liver segmentectomy specimen showed a 3 × 2 × 2.4 cm white nodule puckering the liver capsule.

### Microscopic pathology and immunohistochemistry profile

**Figure 2:**
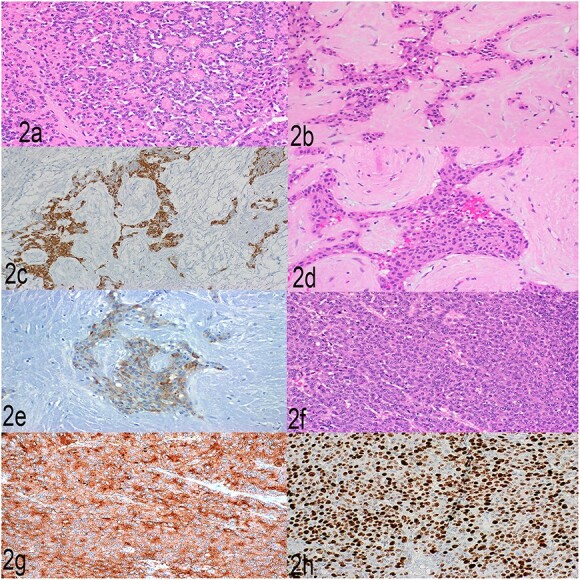
(a) Well-differentiated acinar cells, H&E, 2×. (b) Well-differentiated neuroendocrine tumor, H&E 20×. (c) Well-differentiated neuroendocrine tumor, synaptophysin, 20×. (d) Squamoid nests, H&E, 20×. (e) Squamoid nests, EMA, 20×. (f) LCNEC, H&E, 20×. (g) LCNEC, synaptophysin, 20×. (h) LCNEC, Ki67, 20×.

Histopathological examination showed a large pancreatic tumor with extensive spleen involvement, displaying heterogeneous tumor cell differentiation with both low-grade and high-grade areas (Fig. 1b). Low-grade components included well-differentiated acinar cells in acinar formation (Fig. 2a) and well-differentiated neuroendocrine cells in trabecular formation (Fig. 2b). Neuroendocrine tumor cells were strongly positive for synaptophysin (Fig. 2c). Intermixed with well-differentiated acinar cells were clusters of squamoid cells (Fig. 2d), highlighted by immunohistochemistry (IHC) for EMA (Fig. 2e). IHC for beta-catenin showed nuclear positivity, similar to that in the previous splenic biopsy. These findings confirmed a diagnosis of pancreatoblastoma.

The high-grade component showed sheets of solid cells with high mitotic activity (40 per 2 mm^2^), nearly 100% Ki67 proliferation index, and strong positivity for synaptophysin (Fig. 2f–h). The cells had abundant cytoplasm, fitting the diagnostic criteria for large cell neuroendocrine carcinoma. One metastatic lymph node showed low- and high-grade neuroendocrine components, and the liver metastasis showed high-grade large cell neuroendocrine carcinoma. The final diagnosis was high-grade progression to large cell neuroendocrine carcinoma within pancreatoblastoma.

## Discussion

Pancreatoblastoma is a rare malignant neoplasm predominantly in children under 10, with a median age of 4–5 years and a slightly higher prevalence in males (male to female ratio of 1.14:1) [2, 6]. It is usually an indolent tumor with non-specific clinical symptoms and often diagnosed incidentally [1, 7]. The clinical presentation in this case included abdominal pain, hepatosplenomegaly, vomiting, early satiety, occasional diarrhea, and weight loss, consistent with prior reports [1, 2, 4, 6, 8]. Pediatric patients typically show high levels of AFP and CEA, with AFP being a reliable marker for preoperative diagnosis, therapeutic efficacy, and recurrence [4, 7, 9]. The patient’s AFP decreased from 357 ng/ml pre-treatment to 30.9 ng/ml post-surgery, supporting AFP as an effective surveillance marker.

The coexistence of large cell neuroendocrine carcinoma within pancreatoblastoma is extremely rare and previously unreported in children. Typically, pancreatoblastoma exhibits multiple differentiation lines and squamoid nests [3]. This case included both low-grade pancreatoblastoma components and high-grade neuroendocrine carcinoma. The high-grade component met the criteria for large cell neuroendocrine carcinoma, with abundant cytoplasm, high mitotic count, and strong neuroendocrine marker positivity. This rare progression could result from the low-grade component evolving into a high-grade form or from synchronous tumor origins. The intermixing of low- and high-grade components suggests progression from the same tumor origin.

## References

[1] Mahdi M , Abu Alnasr M, Almehman BA, et al. A rare case of Pancreatoblastoma in a Pediatric patient. Cureus 2020;12:e6779. 10.7759/cureus.6779. 32015939 PMC6986467

[2] Benoist S , Penna C, Julié C, et al. Prolonged survival after resection of pancreatoblastoma and synchronous liver metastases in an adult. Hepatogastroenterology 2001;48:1340–2. 11677959

[3] Kartal İ. Childhood neuroendocrine tumors of the digestive system: a single center experience. *Medicine (Baltimore)* 2022;101:e28795. 10.1097/MD.0000000000028795. PMC8830841 35147110

[4] Liu T , Zhao T, Shi C, et al. Pancreatoblastoma in children: clinical management and literature review. Transl Oncol 2022;18:101359. 10.1016/j.tranon.2022.101359. 35180620 PMC8857517

[5] Konukiewitz B , Jesinghaus M, Kasajima A, et al. Neuroendocrine neoplasms of the pancreas: diagnosis and pitfalls. Virchows Arch 2022;480:247–57. 10.1007/s00428-021-03211-5. 34647171 PMC8986719

[6] Zhang X , Ni S-J, Wang X-H, et al. Adult pancreatoblastoma: clinical features and imaging findings. Sci Rep 2020;10:11285. 10.1038/s41598-020-68083-2. 32647222 PMC7347875

[7] Défachelles AS , de Lassalle, Boutard P, et al. Pancreatoblastoma in childhood: clinical course and therapeutic management of seven patients. Journal of Medical and Pediatric Oncology 2001;37:47–52. 10.1002/mpo.1162. 11466723

[8] Bartsch D , Hahn SA, Danichevski KD, et al. Mutations of the DPC4/Smad4 gene in neuroendocrine pancreatic tumors. Oncogene 1999;18:2367–71. 10.1038/sj.onc.1202585. 10327057

[9] Patterson KN , Trout AT, et al. Solid pancreatic masses in children: a review of current evidence and clinical challenges. Front Pediatr 2022;10:1–27. 10.3389/fped.2022.966943. PMC9732489 36507125

